# Comparison of A1 and A2A receptor dynamics using FRET based receptor sensors

**DOI:** 10.1186/2193-1801-4-S1-L6

**Published:** 2015-06-12

**Authors:** Carsten Hoffmann, Anette Stumpf

**Affiliations:** University of Wuerzburg, Germany

**Keywords:** Receptor dynamics, adenosine receptor, FRET

Recently published crystal structures of the adenosine A2A receptor in the active and inactive state have revealed static endpoints of the conformational changes associated with the activation process. To investigate the activation dynamics of different adenosine receptor subtypes we used fluorescence resonance energy transfer (FRET) measurements of a modified A1 and A2A receptor construct (A1R, A2AR). Those optical probes where designed by fusion of the cyan fluorescent protein (CFP) to the C-terminus of the receptor and insertion of the fluorescein arsenical hairpin binder (FlAsH) motif into the 3rd intracellular loop. Based on the ligand binding pocket revealed from the crystal structure 10 optical probes including individual mutations were created for each receptor. To compare A1R and A2AR dynamics, we established HEK293 cell lines stably expressing these optical probes and investigated the signal amplitude and the receptor activation kinetics in living cells. We indentified 3 different effects of these mutations. One class causes problems in membrane localization of the A1R but not the A2AR. The 2nd group is involved in binding of the ribose moiety and has stronger effects in the A1R compared to the A2AR. The 3rd class consists of the mutants that are involved in binding of the adenine moiety and have similar effects for adenosine and theophylline binding for the A2AR. Thus, our study provides evidence that amino acids serve different functions within the A1R and A2AR ligand binding pocket. In summary the different signal amplitudes and different activation kinetics are indicative for a different activation behavior of the A1R and A2AR and the data from the receptor mutants support these findings and gives new insight into the A1R- structure.

